# Associations of serum neurofilament and asthma: evidence from the NHANES 2013–2014

**DOI:** 10.3389/fneur.2025.1567158

**Published:** 2025-07-04

**Authors:** Mingming Yang, Xiao Qian, Luqian Zhu, Qiong Xu

**Affiliations:** Hangzhou TCM Hospital Affiliated to Zhejiang Chinese Medical University, Hangzhou, China

**Keywords:** asthma, serum neurofilament (sNfL), biomarker, neural mechanism, NHANES

## Abstract

**Background:**

Neural mechanisms are important in asthma, and serum neurofilament light chain (sNfL) is a biomarker of neuronal damage. This study investigated the correlation between sNfL and asthma.

**Methods:**

This cross-sectional study was based on NHANES data and used multiple logistic regression analysis, subgroup analysis, and smoothed curve fitting to explore the relationship between sNfL and asthma. Covariate selection was validated using the variance inflation factor (VIF).

**Results:**

The study involved 1773 participants. Results showed that Ln-sNfL (OR = 1.39, 95% CI: 1.03–1.88, *p* = 0.0325) was positively associated with asthma. Subgroup analyses showed that age, gender, race, education level, marital status, family income, BMI, alcohol use, smoking, diabetes, hypertension and PAMs did not affect the positive association between sNfL and asthma (*p* > 0.05) (FDR corrected *p* > 0.1). E-value analysis suggested robustness to unmeasured confounding.

**Conclusion:**

There is a positive correlation between sNFL and asthma, and further large-scale and prospective studies are needed to replicate our results and assess the ability of sNfL to predict asthma.

## 1 Introduction

Asthma is a very common chronic disease worldwide (1), key features include airway hyperresponsiveness and changes in airway mucus quantity and quality (2–4). More than 300 million people worldwide suffer from asthma and it causes about 495,000 deaths each year, reflecting the fact that asthma has become a serious public health problem (5, 6). However, the pathogenesis of asthma is not yet fully understood (7). A growing body of research in recent years has shown that neural mechanisms have an important impact on asthma (8–11). Velden et al. (12) demonstrated in their work that neurogenic mechanisms may play a role in the pathophysiology and pathogenesis of asthma and that the neural regulation of the airways may be aberrant in asthmatics. Therefore, further understanding of the impact of neural mechanisms on asthma is essential for asthma prevention and management.

The most prevalent cytoskeletal element of mature neurons is made up of neuron-specific cytoskeletal fibers called neurofilaments (NfL) (13, 14). Neurofilaments (NfL) are composed of three distinct chains: light, medium and heavy, which are discharged into the surrounding environment after axonal injury, of which serum neurofilament light chain (sNfL) is a biomarker of neuronal injury (15, 16). Elevated serum neurofilament light chain (sNfL) levels in patients with multiple sclerosis, Alzheimer’s disease, Parkinson’s and Huntington’s diseases, motor neuron diseases, cerebrovascular accidents, hereditary peripheral neuropathies, and impairments associated with traumatic brain injury (16–21). In recent years, new studies have suggested that sNfL may serve as a biomarker for non-primary neurological disorders, including those associated with perioperative care, human infectious diseases, and intensive care units (22). The linear correlation between sNfL levels and periodontitis was illustrated in a study by Jing et al., which also suggested that sNfL may serve as a biomarker for non-neurologic diseases (23). Ciardullo et al. (24) showed that the association between diabetes and sNfL remains significant. Qaisar et al. (25) showed that serum NfL levels were significantly elevated in patients with COPD, Rosenkranz et al. (26) showed that Alzheimer’s disease and related dementia (ADRD) is more common with asthma, especially when asthma is more severe or worsens more frequently, this suggests an association between sNfL and respiratory disease.

To date, few studies have explored the association between sNfL and asthma in the general population. In this study, the potential association between sNfL and asthma levels was explored through a large-scale survey of US adults. It aims to provide a novel solution for the early prevention of asthma and the exploration of its underlying mechanisms.

## 2 Materials and methods

### 2.1 Survey description

The National Center for Health Statistics (NCHS) performed a countrywide research (NHANES) to evaluate the state of nutrition and health in the United States, which served as the basis for the cross-sectional data. Every survey respondent signed an informed consent form, and the NCHS research Ethics Review Committee authorized all NHANES study protocols. Data on demographics, diet, examinations, lab work, and questionnaires were submitted by the participants. All full NHANES research designs and data are available to the public at www.cdc.gov/nchs/NHANEs/. Strengthening the Reporting of Observational Studies in Epidemiology (STROBE) reporting guidelines were met by the cross-sectional study.

### 2.2 Study population

**Figure 1:**
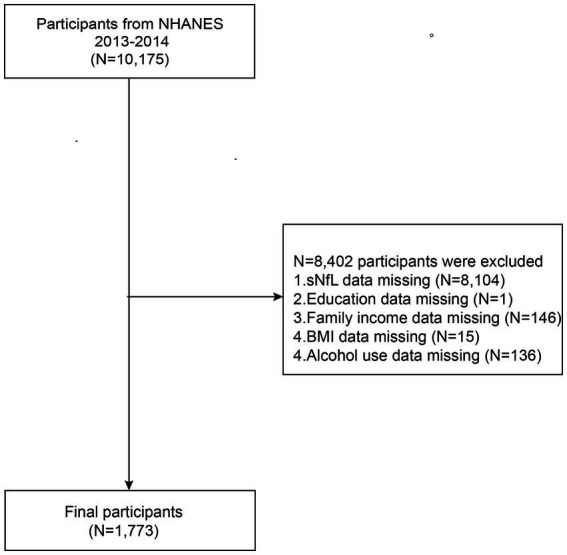
Flowchart of study population inclusion in 2013–2014 NHANES. NHANES, National Health and Nutrition Examination Survey; sNfL, serum neurofilament light chain; Family income, the ratio of family income to poverty; BMI, body mass index.

After a comprehensive search and screening of the NHANES database, participants from 2013 through 2014 were included in this study (sNfL data were not available for other years). Initially, 10,175 participants were recruited. Since the focus of this paper is to examine the relationship between sNfL, and asthma, exclusion criteria for this study included (a) participants with incomplete information on sNfL (*n* = 8,104), (b) participants with incomplete information on family income (*n* = 146) or education(*n* = 1), (c) participants with missing data on BMI (n = 15) or alcohol use (*n* = 136). A total of 1,773 individuals qualified for analysis, as shown in Figure 1.

### 2.3 Assessment of asthma

Due to the limited diagnostic methods for asthma in the NHANES database, referring to other literature on research methods, the definition of current asthma in this paper is a positive response to the following two questions: “Has a doctor or other healthcare professional ever told you that you have asthma?” and “Do you still have asthma?” The control group consists of participants who do not currently have asthma (i.e., those who report never having been diagnosed with asthma by a healthcare professional and those who report having been diagnosed with asthma by a healthcare professional but deny still having asthma). Since the diagnosis of asthma is based on questionnaires, it may introduce recall and misclassification bias.

### 2.4 Assessment of sNfL concentration

Serum neurofilament light chain was the exposure variable in this study, and data were obtained from SSSNFL values in the laboratory data. nHANES’ sNfL assay is as follows: Serum samples for testing NfL concentrations were obtained from individuals between the ages of 20 and 75 years who participated in the 2013–2014 NHANES study and consented to have their samples used for further research. Blood was collected from a vein in the participant’s arm by a trained phlebotomist. The collected blood was divided into vials and stored at a mobile screening center. The vials are then refrigerated or frozen and transported to various laboratories throughout the United States. Siemens Healthineers’ high-throughput acridinium ester (AE) immunoassay on the Atellica immunoassay system was used to measure the amount of sNfL. Following incubation with AE-labeled antibodies that specifically target the NfL antigen, paramagnetic particles coated with capture antibodies were introduced into the blood samples. After unbound AE-labeled antibodies were removed, an acid and a base were used to start a chemiluminescence reaction, which produced detectable light emission. See https://wwwn.cdc.gov/Nchs/Nhanes/2013 2014/SSSNFL_H.htm for more information.

### 2.5 Assessment of covariates of interest

Consistent with previous studies, our multivariate model considered potential covariates that could confound the relationship between sNfL and asthma. Demographic covariates included gender, age, race, education level, marital status, and family income. Body measure covariates included body mass index(BMI). Questionnaire variables also included alcohol use, smoking, diabetes, and hypertension status. Race included Mexican American, other Hispanic, non-Hispanic white, non-Hispanic black, and other races. Marital status was categorized as married/living with partner, widowed/divorced/separated, and never married. Education level includes less than high school, high school, more than high school, and other. Family income was categorized as 0–1.5, 1.5–3.5, >3.5. Drinking status (at least 12 alcoholic beverages in 1 year), smoking status (at least 100 cigarettes in your lifetime), diabetes status (your doctor has told you that you are diabetic), and high blood pressure status (ever said you have high blood pressure) were categorized as yes, no, or other. Considering that prophylactic asthma medications (PAMs) are associated with asthma and may have an impact on the results of the analysis, we included PAMs as covariates in the analysis. PAMs include inhaled corticosteroids, leukotriene receptor antagonists, long-acting *β* agonists, mast cell stabilizers, and theophylline, and treatment with any of these is considered yes and the others are considered no. Individuals can access all comprehensive NHANES research designs and data at www.cdc.gov/nchs/NHANEs/.

### 2.6 Statistical analysis

**Figure 2:**
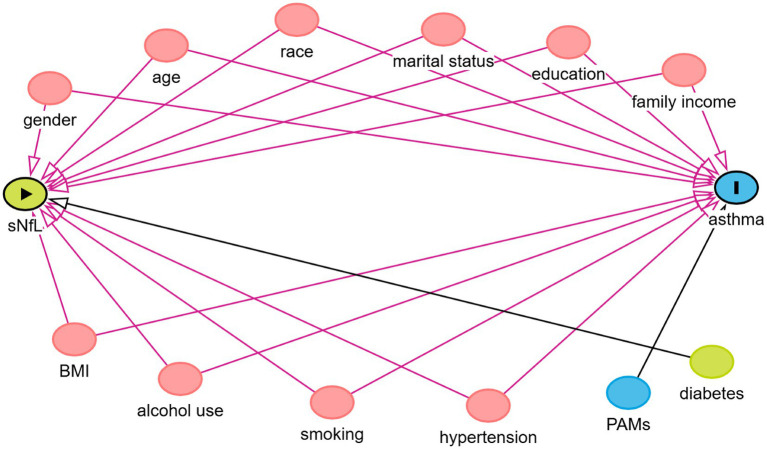
Directed acyclic graphs for the causal effect of sNfL on asthma. Family income, the ratio of family income to poverty; sNfL, serum neurofilament light chain; BMI, body mass index; PAMs, prophylactic asthma medications.

Descriptive statistics included mean with standard deviation (SD) and median with interquartile range (IQR) for continuous data, and frequencies and proportions for categorical data. Consistent with the methodology of previous studies, this study used logistic regression analysis to analyze the risk relationship between sNfL and asthma, and three logistic regression models were developed based on different confounders. Of these, Model 1 was not adjusted for any confounders; Considering that demographic variables are usually important confounding factors affecting many health and behavioral outcomes, and based on previous studies, gender, age and race may have a certain impact on asthma, gender, age and race were included as covariates in Model 2; and in Model 3, in order to solve the problem of multicollinearity and covariate selection, variance expansion factor (VIF) was used for variable selection. A general rule of thumb is that a VIF value >5 indicates problematic covariance (27). The VIF values for gender, age, race, education level, marital status, family income, PAMs, BMI, alcohol use, smoking, diabetes, and hypertension were all calculated to be less than 5, so all the covariates were included. In addition, we used directed acyclic plots^1^ to show the hypothesized relationships between variables (Figure 2).

In this study, we observed that the sNfL data were unevenly distributed and skewed. Considering that logarithmic transformations help stabilize variance and reduce the influence of outliers when dealing with skewed data, we use natural logarithms (Ln)-sNfL, and we check to confirm that all participants have no outliers with sNfL = 0 before transforming. Since the logarithmic transformation changes the values, the clinical significance cannot be directly judged by the magnitude of the values from the clinical point of view, and the relationship between the variables may be changed, and the results need to be carefully interpreted. Therefore, when reporting the results, we provide logistic regression results between sNfL and asthma and directly demonstrate the relationship between sNfL and asthma by smoothing curves to allow the reader to compare and evaluate the effects of the conversions. To assess the robustness of the sensitivity analyses, we conducted a linear trend test using the sNfL interquartile grouping as the dependent variable (28). Subgroup analyses were performed using stratified multivariate logistic regression models stratified by factors including all covariates. In addition, we explored potential unmeasured confounding factors between sNfL and asthma through the calculation of E-values (28, 29). E-values quantify the required magnitude of an unmeasured confounding factor that could invalidate the observed association between sNfL and asthma. Smoothed curve fitting was used in this study to determine if there was a linear relationship between sNfL and asthma. All analyses were performed using EmpowerStats,^2^ PackageR,^3^ and SPSS27.0. In order to ensure the accuracy of the results, this paper does not use multiple interpolation, but excludes the samples with missing variables to ensure that all samples have accurate data in each variable. In determining statistical significance, a two-sided value of *p* < 0.05 was used. In subgroup analyses, the Benjamin-Hochberg method was used to control for false discovery rate (FDR), and an FDR-corrected *p* < 0.1 was considered significant (30, 31).

## 3 Results

### 3.1 Baseline characteristics of participants

A total of 1773 participants with asthma data were enrolled in this study, all over 20 years of age, with a prevalence of asthma of 9.13%. Table 1 is a table of weighted demographic baseline characteristics based on asthma. Asthma was used as a stratification variable. The presence or absence of asthma was significantly associated with sNfL, BMI, hypertension, diabetes, PAMs, gender, race, and marital status (*p* < 0.05). The proportion of sNfL, BMI, hypertension, and smoking was higher in patients with asthma compared with those without asthma, and the prevalence of asthma was higher in women than in men.

**Table 1:** Characteristics of the study population in NHANES by asthma status.

Characteristics	Non-asthma	Asthma	*p*-value
	*N* = 1,611	*N* = 162	
Age(years), median (IQR)	47.00 (26.00)	52.00 (25.75)	0.074
BMI (kg/m^2^), median (IQR)	27.70 (8.25)	31.35 (13.23)	<0.001
sNfL, median (IQR)	12.30(10.50)	13.35 (13.88)	0.024
Ln-sNfL, mean ± SD	2.54 ± 0.65	2.69 ± 0.76	0.024
Gender, *N* (%)			<0.001
Male	811 (50.34%)	44 (27.16%)	
Female	800 (49.66%)	118 (72.84%)	
Race/Ethnicity, N (%)			0.348
Mexican American	225 (13.97%)	16 (9.88%)	
Other Hispanic	143 (8.88%)	14 (8.64%)	
Non-Hispanic White	736 (45.69%)	83 (51.23%)	
Non-Hispanic Black	283 (17.57%)	32 (19.75%)	
Other Race	224 (13.90%)	17 (10.49%)	
Education level, N (%)			0.806
Less than high school	320 (19.86%)	35 (21.60%)	
High school	335 (20.79%)	35 (21.60%)	
More than high school	956 (59.34%)	92 (56.79%)	
Marital status, N (%)			0.021
Married/Living with partner	1,004 (62.32%)	88 (54.32%)	
Widowed/Divorced/Separated	294 (18.25%)	44 (27.16%)	
Never married	313 (19.43%)	30 (18.52%)	
Family income, N (%)			0.074
0–1.5	608 (37.74%)	74 (45.68%)	
1.5–3.5	469 (29.11%)	47 (29.01%)	
>3.5	534 (33.15%)	41 (25.31%)	
Hypertension, N (%)			<0.001
Yes	542 (33.64%)	84 (51.85%)	
No	1,067 (66.23%)	78 (48.15%)	
Other	2 (0.12%)	0 (0.00%)	
Diabetes, N (%)			0.492
Yes	178 (11.05%)	21 (12.96%)	
No	1,384 (85.91%)	134 (82.72%)	
Other	49 (3.04%)	7 (4.32%)	
Smoking, N (%)			0.764
Yes	711 (44.13%)	74 (45.68%)	
No	899 (55.80%)	88 (54.32%)	
Other	1 (0.06%)	0 (0.00%)	
PAMs, N (%)			<0.001
Yes	1,594 (98.94%)	125 (77.16%)	
No	17 (1.06%)	37 (22.84%)	
Alcohol use, N (%)			0.388
Yes	1,209 (75.05%)	114 (70.37%)	
No	399 (24.77%)	48 (29.63%)	
Other	3 (0.19%)	0 (0.00%)	

Categorical variables are described by the number of subjects (percentage); Continuous variables with a normal distribution are described by mean ± SD; Continuous variables without a normal distribution are described by median (IQR). Family income (%), the ratio of family income to poverty; sNfL, serum neurofilament light chain; BMI, body mass index; PAMs, prophylactic asthma medications.

### 3.2 The associations of sNfL and asthma

**Figure 3:**
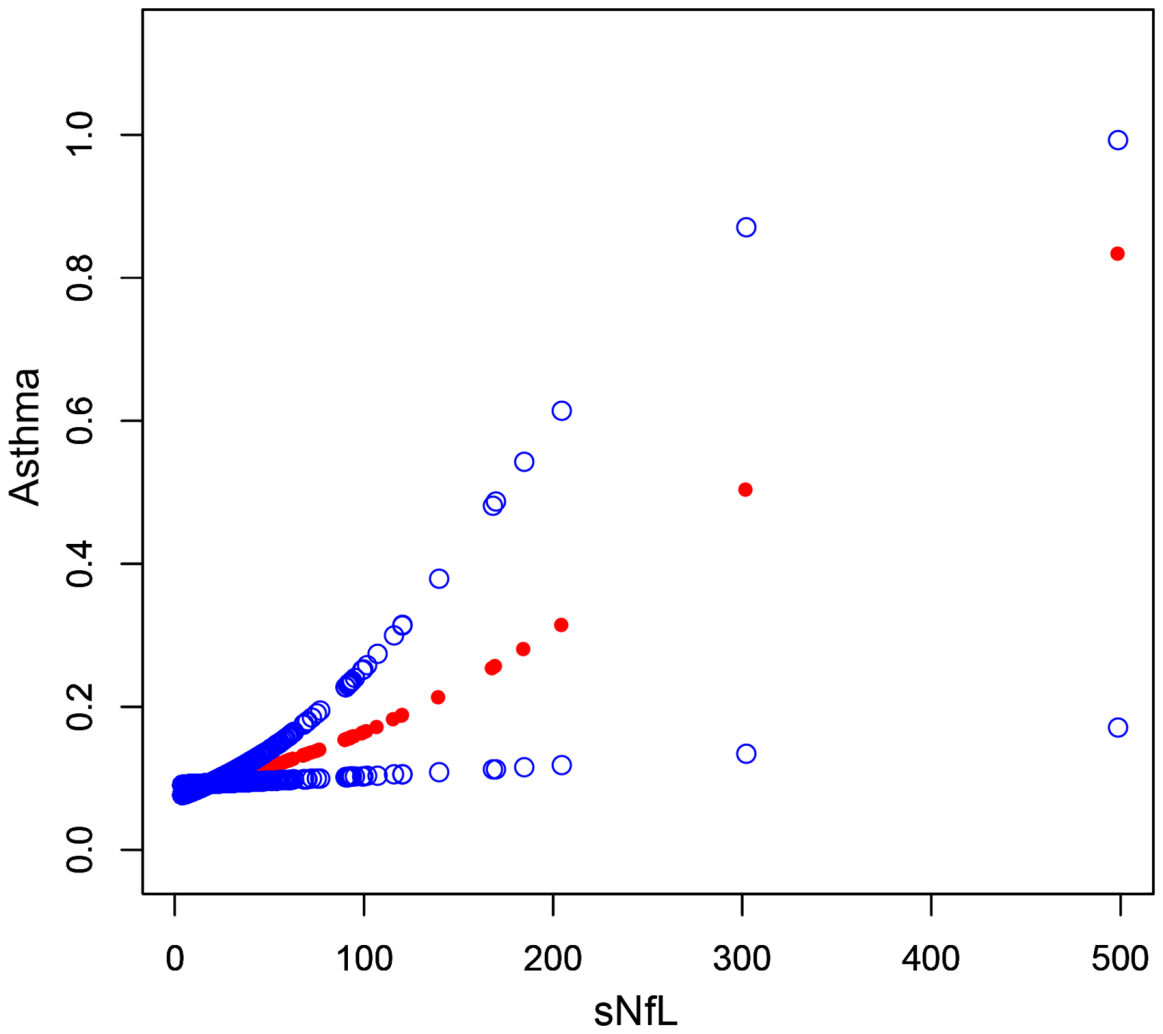
The association between sNfL and asthma. The solid red line represents the smooth curve fit between variables. Blue bands represent the 95% confidence interval from the fit.

**Figure 4:**
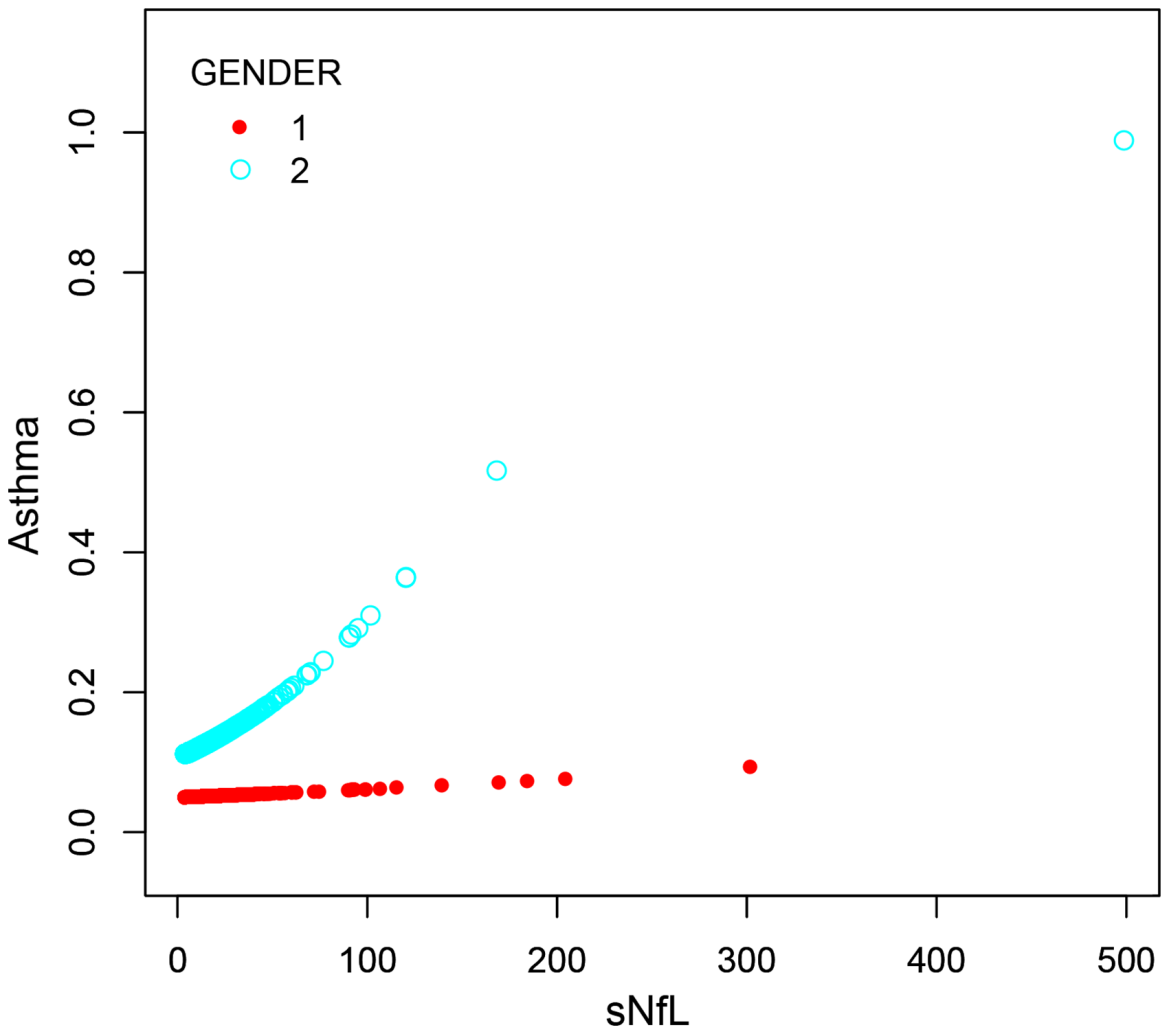
The association between sNfL and asthma stratified by gender.

To assess the association between sNfL and asthma, we performed multivariate logistic regression analyses, which showed that the correlation between Ln-sNfL and asthma remained significant in both partially adjusted and fully adjusted models. As shown in Table 2, each point increase in Ln-sNfL was associated with a 39% increase in asthma prevalence. To further visualize the correlation between sNfL and asthma, a smoothed curve fitting was performed based on Model 3. Smooth curve fitting showed a positive correlation between sNfL and asthma (Figure 3). After stratified analysis by gender, we found that women were more likely to have asthma than men at the same sNfL level (Figure 4).

**Table 2:** Association between sNfL and asthma.

Variable	Crude model (Model 1)	Partially adjusted model (Model 2)	Fully adjusted model (Model 3)
	OR (95% CI) *p*-value	OR (95% CI) *p*-value	OR (95% CI) *p*-value
sNfL	1.01 (1.00, 1.01) 0.0100	1.01 (1.00, 1.01) 0.0209	1.01 (1.00, 1.01) 0.0165
Ln-sNfL	1.36 (1.08, 1.72) 0.0089	1.42 (1.07, 1.87) 0.0136	1.39 (1.03, 1.88) 0.0325
Ln-sNfL quartiles			
Q1	1	1	1
Q2	1.18 (0.73, 1.92) 0.4922	1.25 (0.75, 2.06) 0.3914	1.17 (0.68, 2.00) 0.5663
Q3	1.11 (0.68, 1.80) 0.6739	1.11 (0.64, 1.92) 0.7065	0.95 (0.53, 1.71) 0.8717
Q4	1.60 (1.01, 2.53) 0.0432	1.63 (0.94, 2.82) 0.0814	1.48 (0.82, 2.68) 0.1895

Insensitivity analysis, Ln-sNfL was converted from a continuous variable to a categorical variable (quartiles). OR, odds ratio; Cl, confidence interval; Family income, the ratio of family income to poverty; sNfL, serum neurofilament light chain; BMI, body mass index, PAMs, prophylactic asthma medications. Model 1, no covariates were adjusted. Model 2, age, gender, race were adjusted. Model 3, age, gender, race, education level, marital status, family income, BMI, alcohol use, smoking, diabetes, hypertension and PAMs were adjusted.

### 3.3 Sensitivity analysis

Based on this, subgroup analysis of the relationship between sNfL and asthma (Table 3), age, gender, race, education level, marital status, family income, BMI, alcohol use, smoking, diabetes, hypertension and PAMs did not affect the positive association between sNfL and asthma (*p* > 0.05) (FDR corrected *p* > 0.1). In addition, the result showed an E-value of 2.12, meaning that the observed association could only be fully explained away if an unmeasured confounder had an odds ratio (OR) of at least 2.12 with both sNfL and asthma. This suggests that our main conclusions have a certain degree of robustness.

**Table 3:** Subgroup analysis of the effect of sNfL on asthma.

Subgroups	OR (95% CI), *p*-value	FDR-corrected p for interaction
Gender		0.8454
Male	1.00 (0.99, 1.01) 0.8835	
Female	1.01 (1.00, 1.03) 0.0334	
BMI (kg/m^2^)		0.9655
(0–18.5)	1.00 (0.00, inf.) 1.0000	
[18.5–24)	1.00 (0.97, 1.03) 0.9868	
≥24	1.01 (1.00, 1.02) 0.0239	
Age(years)		0.8454
[20–44)	0.98 (0.94, 1.03) 0.4409	
[45–64)	1.01 (1.00, 1.01) 0.0748	
≥65	1.02 (1.00, 1.04) 0.0147	
Race/Ethnicity		0.9655
Mexican American	1.02 (0.97, 1.08) 0.3963	
Other Hispanic	1.02 (0.95, 1.09) 0.6183	
Non-Hispanic White	1.01 (1.00, 1.02) 0.0404	
Non-Hispanic Black	1.02 (0.99, 1.04) 0.2496	
Other Race	1.00 (0.97, 1.03) 0.8807	
Education level, N (%)		0.9655
Less than high school	0.99 (0.95, 1.03) 0.6206	
High school	1.02 (0.99, 1.04) 0.1480	
More than high school	1.01 (1.00, 1.02) 0.0341	
Marital status, N (%)		0.9655
Married/Living with partner	1.01 (1.00, 1.02) 0.0181	
Widowed/Divorced/Separated	1.00 (0.97, 1.02) 0.7002	
Never married	0.98 (0.91, 1.05) 0.5884	
Family income, N (%)		0.9655
0–1.5	1.01 (0.99, 1.02) 0.3638	
1.5–3.5	1.02 (1.00, 1.03) 0.1116	
>3.5	1.00 (0.97, 1.04) 0.8750	
Diabetes		0.9655
Yes	1.00 (0.99, 1.02) 0.7318	
No	1.01 (1.00, 1.02) 0.0502	
Other	1.95 (0.00, Inf) 0.9993	
Hypertension		0.9655
Yes	1.01 (1.00, 1.01) 0.0358	
No	1.01 (1.00, 1.03) 0.1115	
Other	0.92 (0.00, inf.) 0.9995	
PAMs		0.9655
Yes	1.01 (1.00, 1.01) 0.0320	
No	1.00 (0.88, 1.15) 0.9494	
Smoking		0.9655
Yes	1.01 (1.00, 1.02) 0.0672	
No	1.01 (1.00, 1.02) 0.0336	
Other	0.56 (0.00, inf.) 0.9940	
Alcohol use		0.9655
Yes	1.01 (1.00, 1.02) 0.0667	
No	1.01 (1.00, 1.02) 0.2010	
Other	1.11 (0.00, Inf) 0.9998	

OR, odds ratio; Cl, confidence interval; Family income, the ratio of family income to poverty; BMI, body mass index; PAMs, prophylactic asthma medications.

## 4 Discussion

This study investigated the association between sNfL and asthma in the United States over the age of 20 years. We found that sNfL was positively associated with asthma in both unadjusted and adjusted models, each point increase in Ln-sNfL was associated with a 39% increase in asthma prevalence. Subgroup analyses showed that age, gender, race, education level, marital status, family income, BMI, alcohol use, smoking, diabetes, hypertension and PAMs did not affect the positive association between sNfL and asthma (*p* > 0.05) (FDR corrected *p* > 0.1). After stratified analysis by gender, we found that women were more likely to have asthma than men at the same sNfL level.

To the best of our knowledge, no previous studies have investigated the association between sNfL and asthma, and similarly, there are no current studies that directly suggest that regulating sNfL levels can affect asthma severity. Many scholars have analyzed in depth the influence of neural mechanisms on asthma, and Verleden has shown that although asthma is nowadays considered to be an inflammatory disease of the airways, neural mechanisms are still very important, and that neuropeptides such as substance P and neurokinin A are present in the human airways and give rise to many of the characteristic asthma traits (32), Vafaee et al. showed that asthma is an inflammatory airway disease influenced by neurological and psychological factors, and that psychotherapy or neurorehabilitation reduces inflammation by modulating the activity of neural circuits and the function of brain centers involved in asthma (33). In addition, neurotrophic factors and neuropeptides are key mediators of neuroimmune interactions, and neuroimmune interactions respond to brain signals secreted from airway nerves, which may be the target of many new therapies for asthma (33). Dixon’s study showed that episodes of bronchospasm in asthmatics are triggered by the activation of neurons in the nasal mucosa, reinforcing the idea that neural mechanisms contribute to asthma exacerbations (34).

sNfL is a biomarker of neuronal injury. Liu et al. found a positive correlation between SII and sNfL (35), and Disanto et al. found a significant positive correlation between sNfL and focal inflammatory MRI lesions of the brain and spinal cord (36). Meyer et al. demonstrated an association between elevated levels of inflammatory cytokines (IL-6 and IL-5) and NfL (37). Yao et al. illustrate that NfL is involved in inflammation (38). This suggests a strong relationship between sNfL and inflammation. Current research on sNFL and respiratory diseases primarily focuses on the impact of respiratory diseases on sNFL levels. Ning Zhu et al. found that current smokers often exhibit elevated sNFL levels, suggesting the potential impact of smoke exposure on neural function damage (39). Raoul Sutter et al. have shown that neuronal damage is widespread and significant in patients with severe COVID-19 (40).

The underlying mechanism for the positive association between sNfL and asthma is unclear. In this paper, we speculate that there may be the following reasons, taking into account the results of previous studies. First, neuronal damage may lead to the release of neuropeptides that cause airway inflammation, thereby increasing the risk of asthma. Ding et al. provided direct evidence of neuropeptide release during neuronal injury in their study (41), and Safwat et al. showed that neuropeptides such as substance P (Substance P, SP) are released after traumatic brain injury (TBI) (42). SP induces inflammation in neutrophils by mediating NK1 receptor-induced synthesis of chemokines such as CCL4 and CXCL8 (43), and inflammation can further contribute to asthma (27, 44–46). Additionally, SP interacting with the Mrgprb2 receptor/MrgprX2 (human) on mast cells leads to the release of pro-inflammatory cytokines, chemokines, and the recruitment of immune cells like neutrophils, monocytes, and macrophages (47). NKA, also known as tachykinin, collaborates with SP to promote bronchoconstriction and enhance the antigen presentation function of DCs when combined with the NK2R signal, supporting the immune response (48). So far, SP and NKA have played roles in promoting inflammation and exacerbating asthma (43). Second, the vagus nerve may also play a role in this process. When the vagus nerve is stimulated, it inhibits the inflammatory response (49). Conversely, when the vagus nerve is inactivated, pro-inflammatory cytokines increase, exacerbating pulmonary inflammation. Neuronal damage may lead to vagus nerve dysfunction, which in turn may contribute to the development or exacerbation of asthma. Last, neuronal damage may lead to changes in psychological states, and psychological stress and emotional states can affect airway responsiveness, further contributing to worsening asthma symptoms. Sultana et al. illustrated in their study that mood dysregulation (e.g., in depression) is a long-term consequence of mild traumatic brain injury (TBI) (50), and it has been shown that there is a significant positive correlation between sNfL and depression (51, 52), and mood disorders such as anxiety and depression are associated with worsening asthma symptoms (53, 54). At the same time, since our research method is a cross-sectional study, we cannot conclude that neuronal damage will cause or exacerbate asthma. Considering the complexity of neural mechanisms in asthma, we speculate that the development of asthma may also lead to neuronal damage, which in turn affects sNfL values. Melissa A Rosenkranz et al. have shown that asthma, especially severe asthma, is associated with features of neuroinflammation and neurodegeneration and may be a potential risk factor for nerve damage and cognitive dysfunction (26). Also in our study, we found that adult women are more likely to have asthma than men, which is consistent with many epidemiologic studies, and the reason may be due to the influence of female sex hormones (55, 56). At present, the mechanism of how sex hormones affect asthma is not fully understood, and its biological mechanism can be studied in detail in the future, including how sex hormones affect the immune system and airway responsiveness through multiple pathways.

Although our results show a statistically significant association between Ln-sNfL and asthma, the odds ratio is relatively small (OR = 1.39, 95% CI: 1.03–1.88), suggesting a weak effect size and a potentially limited clinical impact. Therefore, we emphasize that readers should avoid over-extrapolating the relationship between neuronal damage and asthma when interpreting this study. More mechanistic and longitudinal studies are needed to further elucidate the potential linkages. Clinically, we speculate that although sNfL alone is not sufficient as an independent reference for asthma diagnosis, sNfL may play a role in risk stratification or subtype identification of asthma as part of a future multiplex biomarker panel. At the same time, the dynamics of sNfL within individuals may have an indicative effect on asthma progression or treatment response, especially in patients with severe or refractory asthma, and provide clues for further research into the interactions between the nervous system and the immune system, which may contribute to the understanding of asthma-related systemic inflammatory mechanisms.

The strength of our study is that it is based on national data and the results are broadly applicable to the general population in the United States. Regression analyses adjusted for covariates, and the large sample size allowed us to perform sensitivity and subgroup analyses to confirm the robustness of the results. However, there are still some limitations that need to be stated. This is a cross-sectional study, which can only explore the relationship between sNfL and asthma to provide clues for etiologic studies, and cannot verify the causal relationship between disease and etiology. Considering the complexity of neural mechanisms in the development of asthma disease, we suggest that future longitudinal studies combined with Mendelian randomization analysis can further investigate causality, with a focus on differences between different gender groups, especially This will not only help to shed light on the role of sNfL in asthma disorders, but may also provide a theoretical basis for the development of new intervention strategies. Secondly, due to the limited availability of objective diagnostic methods for asthma in the NHANES database, asthma status in this study was based on self-reported questionnaire data, which may introduce recall bias and potential misclassification. Furthermore, serum IgE measurements, which could help distinguish allergic from non-allergic asthma, were not available in the 2013–2014 NHANES cycle. As a result, we were unable to classify asthma subtypes in this analysis. Future studies with more rigorous diagnostic criteria and biomarker data are needed to confirm these findings and explore asthma heterogeneity. Thirdly, since sNfL only has relevant data in 2013 and 2014 because of the relatively small sample size, a larger population study is needed to validate the relationship. Finally, although we adjusted for several important covariates, residual confounding remains a possibility. Factors such as unmeasured environmental exposures, socioeconomic disparities, and asthma-related medication use may still influence the observed association. To address these limitations, future studies should consider establishing comprehensive asthma cohorts with detailed clinical and environmental data. Experimental models, such as animal studies, could also help elucidate the biological mechanisms linking sNfL and asthma phenotypes. Given that NHANES is a dataset based on the United States, due to significant differences in environmental exposures (such as air pollution and allergen distribution), access to medical resources, and asthma diagnosis and management methods across different regions, these factors may influence the observed association between sNfL and asthma. Additionally, studies have shown that sNfL levels can differ among different racial groups (57), so the results of this study on the relationship between sNfL and asthma cannot be directly generalized to populations in various countries and regions, and further research in different countries and ethnicities is needed.

## 5 Conclusion

This study suggests an association between sNfL and asthma, and further large-scale and prospective studies are needed to replicate our results and assess the ability of sNfL to predict asthma.

## Data Availability

Publicly available datasets were analyzed in this study. This data can be found at: www.cdc.gov/nchs/NHANEs/.

## Footnotes

^1^ www.dagitty.net/dags.html

^2^ http://www.empowerstats.com

^3^ http://www.r-project.org
